# Afundamento da haste femoral em artroplastia total do quadril: Comparação entre princípios de fixação

**DOI:** 10.1055/s-0046-1825543

**Published:** 2026-10-06

**Authors:** André N. B. M. de Macedo, Lourenço P. Peixoto, João A. M. Guimarães, Conrado T. Laett

**Affiliations:** 1 Divisão de Ensino e Pesquisa, Instituto Nacional de Traumatologia e Ortopedia, Rio de Janeiro, RJ, Brasil; 2 Programa de Pós-graduação em Ciências da Reabilitação, Centro Universitário Augusto Motta, Rio de Janeiro, RJ, Brasil

**Keywords:** artroplastia do quadril, desenho de prótese, fêmur, ortopedia, prótese de quadril, arthroplasty, replacement, hip, fêmur, hip prosthesis, orthopedic, prosthesis design

## Abstract

**Objetivo:**

Comparar a magnitude do afundamento entre hastes femorais de cunha única (CU) e dupla (CD) em artroplastias totais do quadril (ATQs) primárias e identificar fatores associados a afundamentos clinicamente relevantes.

**Métodos:**

Estudo de coorte retrospectiva com 82 pacientes (164 quadris) submetidos a ATQ bilateral entre 2012 e 2022, cada um recebendo haste tipo I (Corail) e II (Summit), ambas fabricadas pela DePuy Synthes. O afundamento foi mensurado radiograficamente até 6 meses de pós-operatório, sendo considerado clinicamente relevante quando superior a 3 mm. Variáveis radiográficas incluíram o índice de alargamento cortical (IAC) e a taxa de preenchimento do canal (TPC). As análises estatísticas incluíram teste t pareado, regressão logística e correlação de Spearman.

**Resultados:**

O afundamento médio foi de 1,93 ± 1,16 mm nas hastes tipo I e 2,23 ± 1,57 mm nas tipo II ( *p* = 0,181). Afundamentos clinicamente relevantes ocorreram em 18,2% das hastes tipo I e 31,7% das tipo II (OR = 2,07; *p* = 0,050). Observou-se maior afundamento na primeira cirurgia em artroplastias bilaterais ( *p* = 0,006). Não houve correlação significativa entre afundamento e idade, IMC, IAC ou TPC.

**Conclusão:**

As hastes de CD apresentaram maior risco de afundamento clinicamente relevante. Em ATQs bilaterais, o afundamento é mais pronunciado na primeira cirurgia, sem influência detectável de fatores demográficos ou radiográficos.

## Abstract (en)

**Objective:**

To compare the magnitude of subsidence between single- (SW) and double-wedge (DW) femoral stems in primary total hip arthroplasties (THA), and to identify factors associated with clinically relevant subsidence.

**Methods:**

Retrospective cohort study with 82 patients (164 hips) undergoing bilateral THA between 2012 and 2022, each receiving type I (Corail) and type II (Summit) stems. Subsidence was measured radiographically up to 6 months postoperatively and was considered clinically relevant when greater than 3 mm. Radiographic variables included cortical enlargement index (CEI) and canal fill rate (CFR). Statistical analyses included paired t-test, logistic regression, and Spearman correlation.

**Results:**

The mean subsidence was 1.93 ± 1.16 mm in type I stems and 2.23 ± 1.57 mm in type II ( *p* = 0.181). Clinically relevant subsidence occurred in 18.2% of type I stems and 31.7% of type II (OR = 2.07; *p* = 0.050). Greater subsidence was observed in the first surgery in bilateral arthroplasties ( *p* = 0.006). There was no significant correlation between subsidence, age, body mass index (BMI), CEI, or CFR.

**Conclusion:**

The double-wedge stems were associated with a higher risk of clinically relevant subsidence. In bilateral THA, subsidence is more pronounced at first surgery, with no detectable influence of demographic or radiographic factors.

## Introdução

A artroplastia total do quadril (ATQ) é considerada uma das intervenções cirúrgicas mais bem-sucedidas da medicina moderna. As primeiras ATQs datam da década de 1930, com avanços marcantes na década de 1960 graças às inovações implementadas por John Charnley, 1 que introduziu o conceito de baixo atrito articular ao empregar cabeças femorais de pequeno diâmetro articuladas com soquetes de polietileno de alta densidade, fixadas ao fêmur por hastes metálicas cimentadas. Desde então, o estudo das propriedades biomecânicas das próteses tem impulsionado melhorias técnicas, como revestimentos modernos no componente acetabular, superfícies porosas que favorecem a fixação não cimentada e aperfeiçoamentos no desenho das hastes femorais. 1 2 3

O sucesso da ATQ é influenciado por fatores imutáveis, como a morfologia femoral e os hábitos do paciente, 4 além de aspectos cirúrgicos cruciais, como a estabilidade inicial da haste femoral. A falta de estabilidade da haste pode resultar em afundamento, que causa dismetria entre membros, aumenta as chances de soltura asséptica e pode culminar em algia e necessidade de revisão. 5 6 7 O afundamento é observado principalmente nos primeiros 6 meses de pós-operatório, 8 sendo fundamental um acompanhamento radiográfico. Investigações prévias forneceram evidencias que o design colarado, 5 9 contato adequado com a cortical lateral 10 e progressão controlada da descarga de peso 11 contribuem para a redução dos afundamentos maiores que 3 mm, considerados clinicamente relevantes. Novas investigações são necessárias para identificar outros fatores modificáveis que influenciem o afundamento, em especial na população brasileira, fornecendo subsídios para uma prática cirúrgica mais eficaz.

As hastes utilizadas em técnicas não cimentadas de ATQ dividem-se, principalmente, em dois grupos com princípios distintos de fixação. As de cunha única (CU, tipo I), planas no plano anteroposterior e cônicas no médio-lateral, promovem fixação em três pontos e reduzem o contato com a diáfise femoral, minimizando a reabsorção óssea por sobrecarga cortical. As hastes de cunha dupla (CD, tipo II) buscam maior preenchimento metafisário, aumentando a área de suporte. 2 3 Esse aumento na área de suporte, contudo, pode aumentar o atrito e estimular a remodelação óssea, levando a um aumento potencial do afundamento. 12

No presente estudo, a haste Corail (DePuy Synthes) foi classificada como CU (tipo I), conforme Khanuja et al., 3 com base no seu princípio biomecânico predominante de fixação metafisária e transferência de carga, embora apresente geometria proximal alargada nos planos sagital e coronal, e seção transversal quadrangular.

Levando em conta o impacto nos desfechos clínicos, é fundamental avaliar o afundamento em hastes femorais com diferentes princípios de fixação. Assim, este estudo tem como objetivo principal comparar a magnitude do afundamento entre hastes femorais de CU e de DC em ATQs primárias e, como objetivo secundário, analisar fatores de risco para afundamento clinicamente relevante.

## Métodos

Trata-se de coorte retrospectiva com seguimento pós-operatório de 6 meses para o desfecho afundamento da haste femoral. Foram considerados os pacientes submetidos a ATQ na instituição entre março de 2012 e março de 2022, sendo incluídos os pacientes com ATQ bilateral, que tenham recebido haste tipo I (Corail) em um dos quadris e tipo II (Summit – DePuy Synthes) no outro. Foram considerados critérios de inclusão ter entre 18 e 75 anos no momento de ambas as cirurgias, e não ter sido submetido a outras cirurgias no membro inferior. Foram excluídos os pacientes com perda de seguimento radiográfico. O projeto foi previamente aprovado pelo comitê de ética em pesquisa com seres humanos da instituição, sob o CAAE: 68093223.2.0000.5273.

Todos os procedimentos cirúrgicos foram realizados de forma padronizada. A anestesia consistiu em raquianestesia associada a bloqueio periférico do nervo femoral, guiado por eletroestimulação. A profilaxia antibiótica foi iniciada por via endovenosa com cefazolina, administrada 30 minutos antes da incisão cirúrgica. As cirurgias foram realizadas por acesso posterolateral. No pós-operatório, todos os pacientes foram submetidos a protocolo institucional de profilaxia, com cefazolina por 24 horas, assim como de prevenção de eventos tromboembólicos, com heparina de baixo peso molecular por 30 dias.

Foram analisadas as imagens radiográficas da pelve em incidência anteroposterior obtidas na última consulta pré-operatória, no pós-operatório imediato e nas consultas ambulatoriais de 1, 3 e 6 meses de pós-operatório. O afundamento no período de seguimento foi mensurado pela mudança da altura da haste femoral em relação ao medido na imagem do pós-operatórios imediato. Em cada imagem a altura da prótese foi obtida pela distância entre o ombro da haste femoral e o grande trocânter. Foram considerados afundamentos clinicamente relevantes aqueles maiores que 3 mm ao final dos 6 meses, como definido em demais estudos no tema. 10

A qualidade óssea foi avaliada na imagem pré-operatória pelo índice de alargamento cortical (IAC), a razão entre o diâmetro metafisário 2 cm acima do trocânter menor e o diâmetro do istmo. 13 O encaixe do componente femoral foi avaliado na imagem do pós-operatório imediato, utilizando a taxa de preenchimento do canal (TPC) na transição da parte proximal para distal da haste (A), 2 cm da ponta da haste (C), e no ponto médio entre os pontos anteriores (B). 13 Todas as medidas radiográficas foram feitas com auxílio do software Vivace Connect 2022. Data das cirurgias, sexo e Índice de Massa Corporal (IMC) foram obtidos por análise do prontuário.

De acordo com o teste de Shapiro-Wilk, o afundamento e a idade apresentaram distribuição normal, e foram expressos como média ± desvio padrão (DP), enquanto as demais variáveis foram apresentadas como mediana intervalo interquartil (IIQ). Comparações no afundamento ao final dos seis meses entre tipos de haste e ordem das cirurgias foram feitos com teste t pareado. Efeito nas chances de ocorrência de afundamentos clinicamente relevantes foram avaliados por razão de chance obtidas em regressão logística univariada. Correlações entre as variáveis foram avaliadas com índice de correlação de Spearman. Todas as análises foram realizadas em Python 3.10 (Python Software Foundation) com nível de significância de 0,05.

## Resultados

Ao todo, foram incluídos na amostra 82 pacientes, totalizando 164 quadris analisados, com as características apresentadas na Tabela 1 . Não há indícios que o IMC mude entre a 1ª e a 2ª cirurgias ( *p* = 0,459).

**Tabela 1:** Características demográficas da amostra

Sexo	
Masculino	43 (52%)
Feminino	39 (48%)
**Idade (anos)**	54 ± 10
**Tempo entre cirurgias (meses)**	32 (25–58)
**IMC**	
1 ^a^ cirurgia	29 (26–33)
2 ^a^ cirurgia	28 (25–32)

**Abreviação:** IMC, Índice de Massa Corporal.

Na primeira cirurgia 21 pacientes receberam próteses tipo I, tendo os 61 pacientes restantes recebido próteses tipo II. Foi observado um afundamento de 2,36 ± 1,45 mm ao final de 6 meses após a primeira cirurgia e de 1,79 ± 1,27 mm após a segunda, indício de que o afundamento é maior na primeira cirurgia ( *p* = 0,006). Afundamento clinicamente relevante foi observado em 28 (34,1%) hastes na primeira cirurgia e em 13 (15,8%) na segunda, o que representa uma chance 2,75 vezes maior de ocorrência na primeira cirurgia ( *p* = 0,008).

O afundamento observado ao final dos 6 meses de seguimento foi de 1,93 ± 1,16 mm nas próteses tipo I e 2,23 ± 1,57 mm nas tipo II, o que não oferece indícios de maiores afundamentos em algum dos tipos ( *p* = 0,181). Contudo, foram observados afundamentos clinicamente relevantes em 15 (18,2%) hastes do tipo I e em 26 (31,7%) do tipo II, indicando que as chances de afundamento clinicamente relevante são 2,07 vezes maiores nas próteses do tipo II ( *p* = 0,050).

Os índices radiográficos estão apresentados na Tabela 2 . Não foram observados indícios de correlação do afundamento com IAC, TPC, idade ou IMC em nenhum dos tipos de haste. Também não foram identificadas correlações entre essas variáveis e o afundamento observado, separadamente, na primeira e segunda cirurgia.

**Tabela 2:** Características radiográficas dos quadris

	Tipo I	Tipo II
**IAC**	3,5 (2,9–3,8)	3,3 (3,1–3,9)
**TPC (A)**	0,77 (0,69–0,82)	0,77 (0,70–0,85)
**TPC (B)**	0,79 (0,71–0,85)	0,81 (0,69–0,86)
**TPC (C)**	0,63 (0,56–0,74)	0,76 (0,63–0,82)

**Abreviações:** IAC, índice de alargamento cortical; TPC, taxa de preenchimento de canal.

## Discussão

O principal achado do estudo, pioneiro na avaliação do afundamento das hastes femorais na população brasileira, é o indício de que próteses do tipo II apresentam maiores chances de sofrer afundamentos clinicamente relevantes. Curiosamente, observou-se que ocorrem maiores afundamentos durante a primeira cirurgia, em indivíduos submetidos a ATQ bilateralmente. Não foram encontrados indícios de correlação do afundamento com características demográficas ou avaliações radiográficas.

O risco de afundamento nas próteses tipo II observado aqui é confirmado por outros trabalhos. Em estudo conduzido no hospital geral de veteranos de Taiwan, observou-se incidência de 4,8% de afundamentos clinicamente relevantes nas próteses do tipo II, contra apenas 2,0% nas próteses do tipo I. 13

No Kyushu University Hospital, Japão, observou-se afundamento médio das hastes do tipo I de 0,24 mm, contra 0,65 mm nas hastes do tipo II. Nesse período, afundamento clinicamente relevante foi observado em 10% das hastes do tipo II e em apenas 1% do tipo I. 12 Em ambos os estudos, as taxas de afundamento clinicamente relevante parecem menores do que as observadas no presente trabalho, possivelmente em virtude da diferença entre os contextos institucionais. Instituições terciárias e de referência, como a deste estudo, tendem a atender casos de maior complexidade.

Nesse sentido, os achados aqui apresentados reforçam a possível vantagem biomecânica das hastes tipo I, mesmo em populações mais desafiadoras. Essa vantagem também é sugerida por dados do ‘Total Joint Replacement Registry’ dos Estados Unidos, que observou uma taxa de revisão de 0,5% em 10 anos de seguimento nas hastes tipo I, em contraste com uma taxa de 1,5% nas hastes do tipo II em um banco de dados com mais de 40 mil próteses. 14

Em contraste com os achados anteriores, uma análise retrospectiva de mais de 5 mil ATQs, registradas em um banco de dados regional italiano, não encontrou diferenças significativas de afundamento entre hastes do tipo I e II. 15 Tal divergência poderia ser usada para ilustrar a chamada crise de reprodutibilidade nas ciências médicas, 16 mas é fundamental considerar a hipótese de que a magnitude do efeito estudado, a relação entre princípio de fixação e afundamento da haste, neste caso, varia de acordo com o contexto clínico e populacional. Tendo em vista tal precaução, decisões técnicas, políticas públicas e diretrizes clínicas devem ser fundamentadas em dados gerados no ambiente onde serão aplicadas.

Nesse sentido, as análises apresentadas aqui oferecem suporte à tomada de decisão de cirurgiões e gestores do sistema único de saúde brasileiro. Além disso, este trabalho evidencia que estratégias metodológicas que aumentem a sensibilidade das análises, como o pareamento intraindividual utilizado neste estudo, são essenciais para detectar efeitos sutis, como o benefício observado aqui nas hastes do tipo I, que poderiam passar despercebidos em outros delineamentos.

A análise forneceu um achado inesperado, indicando que indivíduos submetidos a ATQ bilateral, em momentos distintos, tendem a apresentar maiores afundamentos na primeira cirurgia. Compreender as causas é fundamental para estabelecer estratégias eficazes para mitigar esse efeito. Não observamos variações significativas no IMC entre as duas cirurgias, o que torna improvável que esse fator explique a diferença observada no afundamento. O tempo entre as cirurgias parece pequeno, em comparação à variabilidade de idade observada na amostra, de forma que a idade não parece explicar o achado.

Pacientes submetidos a ATQ primária unilateral apresentam aumento no nível de atividade física nos anos seguintes à cirurgia. 17 Contudo, é possível que o mesmo não ocorra após a segunda cirurgia nos casos bilaterais. Assim, investigações futuras que avaliem o nível de atividade física, e sua relação com o afundamento da haste em casos bilaterais, podem ser esclarecedoras. Tendo em vista que o nível de atividades físicas intensas parece aumentar as chances de revisão em 10 anos, 18 tal investigação pode ajudar ainda a entender as causas de falha nos casos unilaterais.

Nesse estudo não foram encontrados indícios de que qualidade óssea, encaixe da haste ou características demográficas tenham associação com o afundamento da haste, em nenhum dos tipos avaliados. Embora algumas investigações tenham encontraram correlações fracas de IMC e idade, 19 ou IMC, 8 com afundamento da haste, diversas outras não encontraram qualquer correlação significativa. 12 13 18 20 21

Coletivamente, esses achados sugerem que, embora possa haver algum grau de influência das características do paciente sobre o afundamento, esse efeito é pequeno e dificilmente detectado. É possível que essa dificuldade esteja relacionada com o erro da medida de afundamento obtida por radiografia. Esse erro é evidenciado pelos relatos de afundamento negativos e casos em que a haste parece se afastar do fêmur, observados em todos os estudos citados acima, inclusive o presente. Dada essa baixa potência estatística, não é surpreendente que os estudos que encontraram correlações tenham ao menos 300 próteses avaliadas. Os achados do presente estudo reforçam que mesmo nos contextos em que o efeito das características radiográficas do fêmur proximal e demográficas do paciente não podem ser detectados, o princípio de fixação da haste é um determinante mecânico importante do afundamento.

Esse trabalho oferece contribuição inédita ao investigar a relação entre princípios de fixação da haste femoral e afundamento em serviço de referência no tratamento ortopédico de alta complexidade do sistema único de saúde brasileiro. Além disso, o estudo identificou maiores afundamentos na primeira cirurgia em casos bilaterais, um achado aparentemente inédito.

Contudo, o estudo apresenta limitações importantes. A principal delas refere-se à utilização de radiografias bidimensionais para mensuração do afundamento, técnica sujeita a erros de posicionamento e sobreposição anatômica. Apesar da vantagem metodológica de incluir pacientes submetidos a ATQ bilateral, o delineamento não foi balanceado. A distribuição entre tipo de haste e ordem da cirurgia não foi controlada, o que dificulta a distinção entre os efeitos desses dois fatores.

Cabe ressaltar que tal balanceamento, embora metodologicamente desejável, implicaria em selecionar o tipo de haste com base no interesse investigativo e não nos critérios clínicos, o que seria eticamente inaceitável. É importante considerar que as hastes Corail utilizadas, embora consistentes com o princípio de fixação característico das tipo I, apresentam diferenças geométricas relevantes em relação a outras hastes dessa categoria. Assim, os resultados deste estudo podem não ser plenamente generalizáveis para todas as próteses do tipo I.

## Conclusão

As hastes tipo II apresentam maiores riscos de afundamentos clinicamente relevantes nos primeiros 6 meses de pós-operatório de ATQ primaria. Em artroplastias bilaterais, o afundamento é maior na primeira cirurgia. Não foram encontradas correlações entre o afundamento da haste e características radiográficas do fêmur proximal ou características demográficas do paciente, independentemente do princípio de fixação da haste.
